# (*E*)-Isopentyl 3-(3,4-dihy­droxy­phen­yl)­acrylate

**DOI:** 10.1107/S1600536812003352

**Published:** 2012-02-04

**Authors:** Shuang-Shuang Gu, Jun Wang, Fei Pan, Na Pang, Fu-An Wu

**Affiliations:** a School of Biological and Chemical Engineering, Jiangsu University of Science and Technology, Zhenjiang 212018, People’s Republic of China; b Sericultural Research Institute, Chinese Academy of Agricultural Sciences, Zhenjiang 212018, People’s Republic of China

## Abstract

The title compound, C_14_H_18_O_4_, a derivative of caffeic acid, has an *E* configuration about the C=C bond. The benzene ring is almost coplanar with the C=C—C(O)—O—C linker [maximum deviation = 0.050 (2) Å], making a dihedral angle of only 4.53 (2)°. In the mol­ecule, the adjacent hy­droxy groups form an O—H⋯O inter­action. In the crystal, mol­ecules are linked by O—H⋯O hydrogen bonds, generating a chain propagating in the [110] direction.

## Related literature

For the biological properties of caffeic acid esters, see: Buzzi *et al.* (2009 ▶); Uwai *et al.* (2008 ▶). For synthetic details, see: Feng *et al.* (2011 ▶); Wang *et al.* (2011 ▶). For related structures, see: Xia *et al.* (2004 ▶, 2006 ▶); Wang *et al.* (2011 ▶).
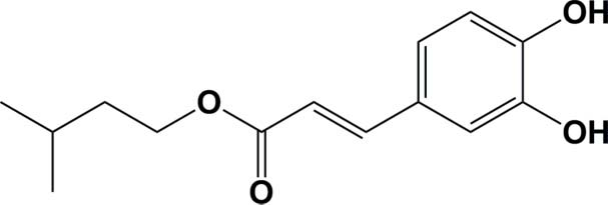


## Experimental

### 

#### Crystal data

C_14_H_18_O_4_
*M* *_r_* = 250.28
Triclinic, 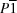

*a* = 5.2790 (11) Å
*b* = 10.244 (2) Å
*c* = 13.834 (3) Å
α = 69.05 (3)°
β = 80.11 (3)°
γ = 78.79 (3)°
*V* = 681.0 (2) Å^3^
*Z* = 2
Mo *K*α radiation
μ = 0.09 mm^−1^
*T* = 293 K
0.30 × 0.20 × 0.10 mm

#### Data collection

Enraf–Nonius CAD-4 diffractometer
Absorption correction: ψ scan (North *et al.*, 1968 ▶) *T*_min_ = 0.974, *T*_max_ = 0.991
2507 measured reflections
2507 independent reflections
1300 reflections with *I* > 2σ(*I*)
3 standard reflections every 200 min intensity decay: 1%

#### Refinement

*R*[*F* ^2^ > 2σ(*F* ^2^)] = 0.059
*wR*(*F* ^2^) = 0.156
*S* = 1.00
2507 reflections
163 parameters
2 restraints
H-atom parameters constrained
Δρ_max_ = 0.14 e Å^−3^
Δρ_min_ = −0.14 e Å^−3^

### 

Data collection: *CAD-4 EXPRESS* (Enraf–Nonius, 1994 ▶); cell refinement: *CAD-4 EXPRESS*; data reduction: *XCAD4* (Harms & Wocadlo, 1995 ▶); program(s) used to solve structure: *SHELXS97* (Sheldrick, 2008 ▶); program(s) used to refine structure: *SHELXL97* (Sheldrick, 2008 ▶); molecular graphics: *SHELXTL* (Sheldrick, 2008 ▶); software used to prepare material for publication: *PLATON* (Spek, 2009 ▶).

## Supplementary Material

Crystal structure: contains datablock(s) I, global. DOI: 10.1107/S1600536812003352/su2370sup1.cif

Structure factors: contains datablock(s) I. DOI: 10.1107/S1600536812003352/su2370Isup2.hkl

Supplementary material file. DOI: 10.1107/S1600536812003352/su2370Isup3.cml

Additional supplementary materials:  crystallographic information; 3D view; checkCIF report

## Figures and Tables

**Table 1:** Hydrogen-bond geometry (Å, °)

*D*—H⋯*A*	*D*—H	H⋯*A*	*D*⋯*A*	*D*—H⋯*A*
O4—H4*A*⋯O3	0.82	2.28	2.721 (2)	114
O3—H3*A*⋯O1^i^	0.82	1.95	2.764 (2)	173
O4—H4*A*⋯O3^ii^	0.82	2.13	2.831 (2)	143

Symmetry codes: (i) 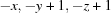; (ii) 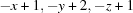.

**Fig. 1.**
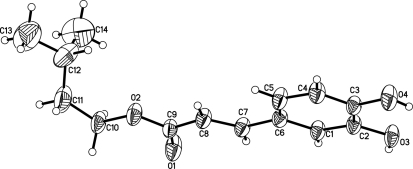
The molecular structure of the title molecule, with the atom numbering scheme and displacement ellipsoids drawn at the 30% probability level.

**Fig. 2.**
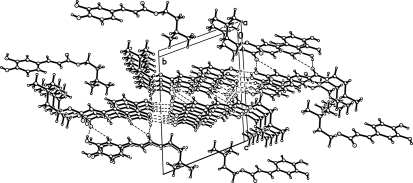
A view along the a-axis of the crystal packing of the title compound. Hydrogen bonds are shown as dashed lines - see Table 1 for details.

## supplementary crystallographic information

### Comment

Caffeic acid esters are a component of propolis (a vegetable resin) and are reported to have a broad spectrum of biological effects, such as, anti-tumour, antioxidant, and anti-inflammatory activities (Uwai *et al.*, 2008; Buzzi *et al.*, 2009). The resin itself has been used as a cation-exchange resin for heterogeneous catalyst (Feng *et al.*, 2011). This prompted us to synthesize a series of caffeic acid esters to investigate their properties better (Wang *et al.*, 2011). Herein, we report on the crystal structure of the title compound, the isopentyl derivative of caffeic acid.

The title molecule has an *E* configuration about the C7═C8 bond (Fig. 1). The benzene ring with the C7═C8—C9 linker is almost coplanar, with a root mean square deviation from the mean plane of 0.005 Å. All bond lengths and angles are in very close agreement with those found in similar caffeic acid structures (Xia *et al.*, 2004, 2006), and in the pentyl derivative of caffeic acid (Wang *et al.*, 2011).

In the crystal, the hydroxy groups contribute to intermolecular O—H···O interactions (Table 1), that link the molecules into ribbons extending in the [110] direction (Fig. 2). On the other hand, the intramolecular O—H···O H-bond also contributes to the stability of the molecular configuration (Fig. 1 and Table 1).

### Experimental

The synthesis follows the method of (Wang *et al.*, 2011). Esterification of caffeic acid with hexyl alcohol was performed in a column (inner diameter = 15 mm, length = 200 mm). A cation exchange resin CD-552 particles (5 g) molecular sieve (5 g) and glass beads of 2 mm in diameter were packed into the middle of the reactor. In a reaction mixture tank, 9 g of caffeic acid was mixed with 100 ml hexyl alcohol. The reaction mixture was supplied to the reaction column at a aret of 10.0 ml/h. The reaction continued at 353 K for 24 h. The solvent was then removed under reduced pressure. The residue was extracted with ethyl acetate three times and filtered. The filtrate was washed successively with dilute saturated aqueous NaHCO_3_ solution, saturated aqueous NaCl, then dried over MgSO_4_, and evaporated. The residue was recrystallized from ethanol to give the title compound as colourless crystals (Yield 5.2 g; 57.7%).

### Refinement

The OH and C-bound H-atoms were included in calculated positions and treated as riding atoms: O-H = 0.82 Å, C-H = 0.93, 0.98, 0.97 and 0.96 Å for CH(aromtic), CH, CH_2_, and CH_3_ H-atoms, respectively, with U_iso_(H) = k × U_eq_(O,C), where k = 1.5 for OH and CH_3_ H-atoms, and k = 1.2 for all other H-atoms.

### Crystal data

**Table:**

C_14_H_18_O_4_	*Z* = 2
*M**_r_* = 250.28	*F*(000) = 268
Triclinic, *P*1	*D*_x_ = 1.220 Mg m^−^^3^
Hall symbol: -P 1	Mo *K*α radiation, λ = 0.71073 Å
*a* = 5.2790 (11) Å	Cell parameters from 25 reflections
*b* = 10.244 (2) Å	θ = 9–13°
*c* = 13.834 (3) Å	µ = 0.09 mm^−^^1^
α = 69.05 (3)°	*T* = 293 K
β = 80.11 (3)°	Block, colourless
γ = 78.79 (3)°	0.30 × 0.20 × 0.10 mm
*V* = 681.0 (2) Å^3^	

### Data collection

**Table:**

Enraf–Nonius CAD-4 diffractometer	1300 reflections with *I* > 2σ(*I*)
Radiation source: fine-focus sealed tube	*R*_int_ = 0.000
Graphite monochromator	θ_max_ = 25.4°, θ_min_ = 1.6°
ω/2θ scans	*h* = −6→6
Absorption correction: ψ scan (North *et al.*, 1968)	*k* = −11→12
*T*_min_ = 0.974, *T*_max_ = 0.991	*l* = 0→16
2507 measured reflections	3 standard reflections every 200 min
2507 independent reflections	intensity decay: 1%

### Refinement

**Table:**

Refinement on *F*^2^	Primary atom site location: structure-invariant direct methods
Least-squares matrix: full	Secondary atom site location: difference Fourier map
*R*[*F*^2^ > 2σ(*F*^2^)] = 0.059	Hydrogen site location: inferred from neighbouring sites
*wR*(*F*^2^) = 0.156	H-atom parameters constrained
*S* = 1.00	*w* = 1/[σ^2^(*F*_o_^2^) + (0.070*P*)^2^] where *P* = (*F*_o_^2^ + 2*F*_c_^2^)/3
2507 reflections	(Δ/σ)_max_ < 0.001
163 parameters	Δρ_max_ = 0.14 e Å^−^^3^
2 restraints	Δρ_min_ = −0.14 e Å^−^^3^

### Special details

**Table:**

Geometry. All e.s.d.'s (except the e.s.d. in the dihedral angle between two l.s. planes) are estimated using the full covariance matrix. The cell e.s.d.'s are taken into account individually in the estimation of e.s.d.'s in distances, angles and torsion angles; correlations between e.s.d.'s in cell parameters are only used when they are defined by crystal symmetry. An approximate (isotropic) treatment of cell e.s.d.'s is used for estimating e.s.d.'s involving l.s. planes.
Refinement. Refinement of *F*^2^ against ALL reflections. The weighted *R*-factor *wR* and goodness of fit *S* are based on *F*^2^, conventional *R*-factors *R* are based on *F*, with *F* set to zero for negative *F*^2^. The threshold expression of *F*^2^ > σ(*F*^2^) is used only for calculating *R*-factors(gt) *etc*. and is not relevant to the choice of reflections for refinement. *R*-factors based on *F*^2^ are statistically about twice as large as those based on *F*, and *R*- factors based on ALL data will be even larger.

### Fractional atomic coordinates and isotropic or equivalent isotropic displacement parameters (Å^2^)

**Table:**

	*x*	*y*	*z*	*U*_iso_*/*U*_eq_	
C1	0.2966 (5)	0.6252 (2)	0.56768 (18)	0.0557 (7)	
H1A	0.1700	0.6372	0.5248	0.067*	
O1	−0.0024 (4)	0.16077 (18)	0.62733 (15)	0.0877 (7)	
O2	0.1887 (4)	0.01321 (17)	0.76260 (13)	0.0743 (6)	
C2	0.4057 (5)	0.7386 (2)	0.55959 (18)	0.0543 (7)	
O3	0.3407 (3)	0.87217 (15)	0.49143 (12)	0.0658 (6)	
H3A	0.2404	0.8698	0.4531	0.099*	
C3	0.5921 (5)	0.7242 (2)	0.62374 (18)	0.0561 (7)	
O4	0.7014 (4)	0.83462 (17)	0.62142 (14)	0.0793 (7)	
H4A	0.6432	0.9064	0.5765	0.119*	
C4	0.6631 (5)	0.5938 (2)	0.6955 (2)	0.0663 (8)	
H4B	0.7863	0.5837	0.7393	0.080*	
C5	0.5533 (5)	0.4770 (3)	0.70355 (19)	0.0666 (8)	
H5A	0.6029	0.3894	0.7523	0.080*	
C6	0.3678 (5)	0.4917 (2)	0.63792 (17)	0.0530 (7)	
C7	0.2509 (5)	0.3738 (2)	0.63921 (18)	0.0570 (7)	
H7A	0.1330	0.3959	0.5909	0.068*	
C8	0.2875 (5)	0.2395 (2)	0.69908 (18)	0.0610 (7)	
H8A	0.4077	0.2102	0.7472	0.073*	
C9	0.1449 (5)	0.1375 (2)	0.69123 (18)	0.0541 (7)	
C10	0.0528 (7)	−0.0973 (3)	0.7622 (2)	0.0821 (10)	
H10A	0.1149	−0.1228	0.7003	0.099*	
H10B	−0.1322	−0.0639	0.7620	0.099*	
C11	0.1015 (8)	−0.2212 (3)	0.8563 (2)	0.1123 (13)	
H11A	0.0061	−0.2928	0.8556	0.135*	
H11B	0.2847	−0.2584	0.8493	0.135*	
C12	0.0392 (9)	−0.2063 (4)	0.9575 (2)	0.1090 (13)	
H12A	0.1470	−0.1399	0.9599	0.131*	
C13	0.1016 (11)	−0.3421 (5)	1.0451 (3)	0.171 (2)	
H13A	0.2824	−0.3785	1.0352	0.256*	
H13B	−0.0021	−0.4098	1.0459	0.256*	
H13C	0.0644	−0.3247	1.1102	0.256*	
C14	−0.2487 (11)	−0.1421 (5)	0.9771 (4)	0.179 (2)	
H14A	−0.2887	−0.0562	0.9206	0.268*	
H14B	−0.2751	−0.1222	1.0411	0.268*	
H14C	−0.3602	−0.2082	0.9815	0.268*	

### Atomic displacement parameters (Å^2^)

**Table:**

	*U*^11^	*U*^22^	*U*^33^	*U*^12^	*U*^13^	*U*^23^
C1	0.0686 (18)	0.0450 (14)	0.0592 (15)	−0.0190 (13)	−0.0249 (13)	−0.0107 (12)
O1	0.1255 (19)	0.0556 (11)	0.0918 (14)	−0.0350 (11)	−0.0638 (14)	−0.0015 (10)
O2	0.1147 (17)	0.0505 (11)	0.0639 (11)	−0.0347 (10)	−0.0391 (11)	−0.0014 (9)
C2	0.0689 (18)	0.0438 (14)	0.0547 (14)	−0.0209 (13)	−0.0223 (13)	−0.0083 (12)
O3	0.0876 (14)	0.0435 (10)	0.0711 (11)	−0.0261 (9)	−0.0368 (10)	−0.0037 (8)
C3	0.0700 (18)	0.0471 (14)	0.0576 (14)	−0.0250 (13)	−0.0186 (13)	−0.0116 (12)
O4	0.1004 (16)	0.0569 (11)	0.0944 (14)	−0.0368 (11)	−0.0507 (11)	−0.0107 (10)
C4	0.080 (2)	0.0540 (16)	0.0741 (17)	−0.0224 (14)	−0.0340 (15)	−0.0142 (14)
C5	0.087 (2)	0.0486 (15)	0.0666 (16)	−0.0247 (14)	−0.0390 (15)	−0.0004 (12)
C6	0.0623 (17)	0.0493 (14)	0.0529 (14)	−0.0235 (13)	−0.0112 (12)	−0.0135 (12)
C7	0.0710 (19)	0.0493 (15)	0.0549 (14)	−0.0226 (13)	−0.0237 (13)	−0.0076 (12)
C8	0.079 (2)	0.0495 (15)	0.0574 (15)	−0.0228 (14)	−0.0284 (14)	−0.0048 (12)
C9	0.0681 (19)	0.0418 (14)	0.0534 (14)	−0.0161 (13)	−0.0183 (13)	−0.0078 (12)
C10	0.143 (3)	0.0514 (16)	0.0632 (16)	−0.0484 (17)	−0.0326 (17)	−0.0050 (13)
C11	0.183 (4)	0.069 (2)	0.092 (2)	−0.058 (2)	−0.050 (2)	0.0008 (18)
C12	0.120 (3)	0.126 (3)	0.073 (2)	−0.055 (3)	−0.016 (2)	−0.0006 (19)
C13	0.223 (6)	0.145 (4)	0.123 (3)	−0.028 (4)	−0.040 (3)	−0.009 (3)
C14	0.171 (6)	0.200 (6)	0.160 (5)	−0.019 (4)	−0.014 (4)	−0.060 (4)

### Geometric parameters (Å, º)

**Table:**

C1—C2	1.356 (3)	C7—H7A	0.9300
C1—C6	1.390 (3)	C8—C9	1.444 (3)
C1—H1A	0.9300	C8—H8A	0.9300
O1—C9	1.207 (3)	C10—C11	1.476 (3)
O2—C9	1.310 (3)	C10—H10A	0.9700
O2—C10	1.455 (3)	C10—H10B	0.9700
C2—O3	1.373 (3)	C11—C12	1.439 (5)
C2—C3	1.388 (3)	C11—H11A	0.9700
O3—H3A	0.8200	C11—H11B	0.9700
C3—O4	1.355 (3)	C12—C13	1.505 (5)
C3—C4	1.377 (3)	C12—C14	1.553 (6)
O4—H4A	0.8200	C12—H12A	0.9800
C4—C5	1.390 (3)	C13—H13A	0.9600
C4—H4B	0.9300	C13—H13B	0.9600
C5—C6	1.398 (3)	C13—H13C	0.9600
C5—H5A	0.9300	C14—H14A	0.9600
C6—C7	1.453 (3)	C14—H14B	0.9600
C7—C8	1.324 (3)	C14—H14C	0.9600
			
C2—C1—C6	122.1 (2)	O2—C10—C11	108.5 (2)
C2—C1—H1A	118.9	O2—C10—H10A	110.0
C6—C1—H1A	118.9	C11—C10—H10A	110.0
C9—O2—C10	117.5 (2)	O2—C10—H10B	110.0
C1—C2—O3	124.0 (2)	C11—C10—H10B	110.0
C1—C2—C3	120.1 (2)	H10A—C10—H10B	108.4
O3—C2—C3	115.9 (2)	C12—C11—C10	119.8 (3)
C2—O3—H3A	109.5	C12—C11—H11A	107.4
O4—C3—C4	118.2 (2)	C10—C11—H11A	107.4
O4—C3—C2	122.6 (2)	C12—C11—H11B	107.4
C4—C3—C2	119.1 (2)	C10—C11—H11B	107.4
C3—O4—H4A	109.5	H11A—C11—H11B	106.9
C3—C4—C5	121.0 (2)	C11—C12—C13	113.3 (4)
C3—C4—H4B	119.5	C11—C12—C14	112.5 (4)
C5—C4—H4B	119.5	C13—C12—C14	109.6 (3)
C4—C5—C6	119.7 (2)	C11—C12—H12A	107.0
C4—C5—H5A	120.1	C13—C12—H12A	107.0
C6—C5—H5A	120.1	C14—C12—H12A	107.0
C1—C6—C5	117.9 (2)	C12—C13—H13A	109.5
C1—C6—C7	119.1 (2)	C12—C13—H13B	109.5
C5—C6—C7	122.9 (2)	H13A—C13—H13B	109.5
C8—C7—C6	129.5 (2)	C12—C13—H13C	109.5
C8—C7—H7A	115.3	H13A—C13—H13C	109.5
C6—C7—H7A	115.3	H13B—C13—H13C	109.5
C7—C8—C9	121.4 (2)	C12—C14—H14A	109.5
C7—C8—H8A	119.3	C12—C14—H14B	109.5
C9—C8—H8A	119.3	H14A—C14—H14B	109.5
O1—C9—O2	121.8 (2)	C12—C14—H14C	109.5
O1—C9—C8	125.2 (2)	H14A—C14—H14C	109.5
O2—C9—C8	113.0 (2)	H14B—C14—H14C	109.5
			
C6—C1—C2—O3	−179.9 (2)	C4—C5—C6—C7	177.9 (3)
C6—C1—C2—C3	−1.0 (4)	C1—C6—C7—C8	−179.5 (3)
C1—C2—C3—O4	−177.8 (3)	C5—C6—C7—C8	1.5 (4)
O3—C2—C3—O4	1.2 (4)	C6—C7—C8—C9	177.8 (3)
C1—C2—C3—C4	−0.3 (4)	C10—O2—C9—O1	0.1 (4)
O3—C2—C3—C4	178.7 (2)	C10—O2—C9—C8	179.5 (2)
O4—C3—C4—C5	178.4 (3)	C7—C8—C9—O1	5.2 (4)
C2—C3—C4—C5	0.8 (4)	C7—C8—C9—O2	−174.1 (2)
C3—C4—C5—C6	0.0 (4)	C9—O2—C10—C11	−172.5 (3)
C2—C1—C6—C5	1.7 (4)	O2—C10—C11—C12	56.0 (4)
C2—C1—C6—C7	−177.4 (2)	C10—C11—C12—C13	−179.7 (3)
C4—C5—C6—C1	−1.2 (4)	C10—C11—C12—C14	55.3 (5)

### Hydrogen-bond geometry (Å, º)

**Table:**

*D*—H···*A*	*D*—H	H···*A*	*D*···*A*	*D*—H···*A*
O4—H4*A*···O3	0.82	2.28	2.721 (2)	114
O3—H3*A*···O1^i^	0.82	1.95	2.764 (2)	173
O4—H4*A*···O3^ii^	0.82	2.13	2.831 (2)	143

Symmetry codes: (i) −*x*, −*y*+1, −*z*+1; (ii) −*x*+1, −*y*+2, −*z*+1.
